# Similarity hypothesis: understanding of others with autism spectrum disorders by individuals with autism spectrum disorders

**DOI:** 10.3389/fnhum.2015.00124

**Published:** 2015-03-17

**Authors:** Hidetsugu Komeda

**Affiliations:** ^1^The Hakubi Center for Advanced Research, Kyoto UniversityKyoto, Japan; ^2^Department of Cognitive Psychology in Education, Graduate School of Education, Kyoto UniversityKyoto, Japan

**Keywords:** similarity, self, other, empathy, autism spectrum disorder, vmPFC, fMRI

## Abstract

Individuals with an autism spectrum disorder (ASD) are generally thought to lack empathy. However, according to recent empirical and self-advocacy studies, individuals with ASD identify with others with ASD. Based on mutual understanding, individuals with ASD respond empathically to others with these disorders. Results have shown that typically developing (TD) adults identify with TD fictional characters, and that such identification plays a critical role in social cognition. TD individuals retrieve episodes involving TD individuals faster than they retrieve episodes involving ASD individuals. Individuals with ASD also show a “similarity effect” whereby they retrieve stories involving ASD individuals more effectively when the stories have consistent outcomes than when they have inconsistent outcomes. In this context, I hypothesized that similarities between a perceiver and a target facilitate cognitive processing. This hypothesis was named the “similarity hypothesis”. Perceivers empathize with targets similar to themselves, which facilitates subsequent cognitive processing. Behavioral and neuroimaging studies are reviewed based on the similarity hypothesis.

## Introduction

Autism spectrum disorder (ASD) is diagnosed based on behaviors such as difficulties with communication and social development, repetitive behaviors, and narrowly focused but strong interests (American Psychiatric Association, 2013). Individuals with ASD have difficulty understanding other people’s inner states, and they are generally thought to lack empathy (Baron-Cohen, 1995; Lombardo et al., 2007). However, according to recent empirical (Komeda et al., 2013a) and self-advocacy studies (Dern, 2008; Ayaya and Kumagaya, 2010), individuals with ASD understand others with ASD. Indeed, individuals with ASD have intact empathy if they do not have alexithymic traits (Bird et al., 2010). Moreover, they show empathic responses toward individuals with ASD (Komeda et al., 2015). The APA dictionary (VandenBos, 2007) defines empathy as understanding a person from that person’s frame of reference rather than from one’s own frame of reference so that the other’s perceptions and thoughts are experienced vicariously. Empathy includes at least two aspects: cognitive and affective (Blair, 2005; Shamay-Tsoory et al., 2005, 2007; Shamay-Tsoory and Aharon-Peretz, 2007; Jones et al., 2010; Schwenck et al., 2012). Cognitive empathy involves perspective taking (Eslinger, 1998) and theory of mind (Premack and Woodruff, 1978; Baron-Cohen et al., 1985) and is dependent on several cognitive capacities. Affective empathy refers to the capacity to experience affective reactions in response to the observed experience of others (Davis, 1994; Shamay-Tsoory, 2011).

Humans often have preferences and feel affinity for their in-group over an out-group (Turner, 1982). For example, people tend to be attracted to and more satisfied with interactions involving individuals similar to them (Byrne and Griffitt, 1969; Carli et al., 1991). People also prefer individuals with personality traits similar to their own (Griffitt, 1966, 1969; Deutsch et al., 1991). Additionally, we preferentially remember other people with whom we share an identity (Sporer, 2001), which is called the in-group memory advantage (Rule et al., 2010).

Recent studies on typically developing (TD) adults have shown that similarities between readers and characters in a story play a critical role in social cognition. For example, it is easier for extraverted participants to understand stories about other extraverted people (Komeda et al., 2009). Additionally, extraverted individuals are able to predict the outcomes of other extraverted people’s actions more easily, and neurotic individuals are able to predict the outcomes of other neurotic people’s actions more easily (Komeda et al., 2013b). TD individuals retrieved target sentences about a TD character’s context faster than they retrieved target sentences about an ASD character’s context (Komeda et al., 2013a).

Individuals with ASD also showed a “similarity effect” in that they retrieved ASD-consistent outcomes more efficiently than they retrieved ASD-inconsistent outcomes if the episodes were about individuals with ASD, whereas they did not respond differentially in response to TD-consistent and TD-inconsistent outcomes of episodes about TD individuals (Komeda et al., 2013a). Therefore, similarities between a perceiver and a target facilitate cognitive processing. This prediction is called the *similarity hypothesis*. The similarity hypothesis was originally part of the *reader-protagonist interaction model* in narrative comprehension (Komeda and Kusumi, 2007).

## Reader-Protagonist Interaction Model

We proposed the reader-protagonist interaction model as a framework to integrate discourse comprehension studies with social cognitive neuroscience studies (Komeda and Kusumi, 2007; Komeda, 2010). This model builds a connection between empathy as a virtual experience during story reading and empathy during social interactions as a real experience.

**Figure 1:**
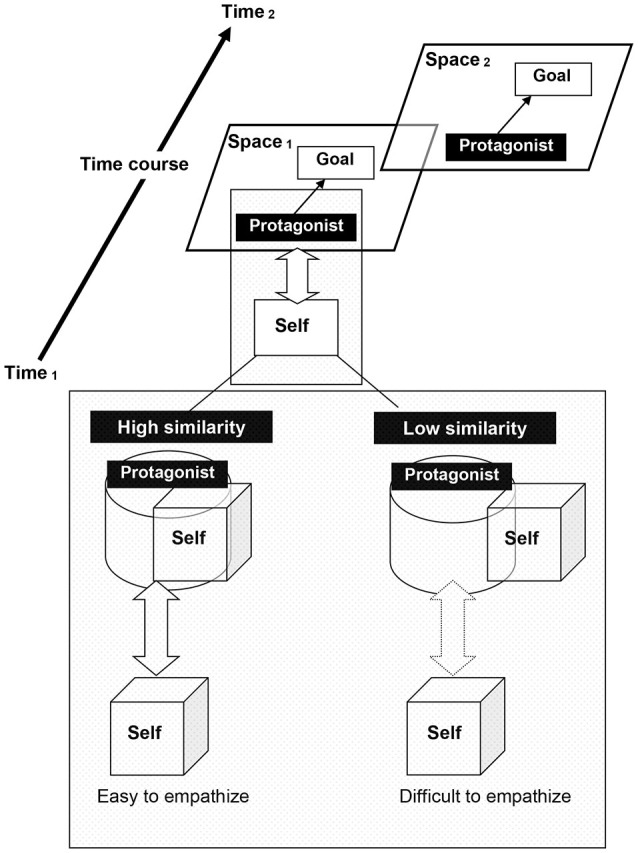
**Reader-protagonist interaction model**. “Self” corresponds to “reader” or “listener” during discourse comprehension. The more the characteristics between self and story protagonists or characters overlap, the greater the similarity.

As shown in Figure 1, mental representations are updated during discourse comprehension when ongoing sentences are mapped on previous contexts (Zwaan and Radvansky, 1998). This mental representation includes spatial and temporal information, story protagonists or characters (or conversational partners), and their goals, motivations, and intentions (Zwaan et al., 1995a,b; Zwaan and Radvansky, 1998; Komeda and Kusumi, 2006). Readers infer and predict the actions of story characters using causal clues described in the situation to understand these actions (van den Broek and Gustafson, 1999; van den Broek et al., 1999). When readers experience causal discontinuities, they feel a sense of strangeness and strive to predict the behavior and mental states of the story character, such as desires, motivations, and feelings (Miall, 1989; Komeda et al., 2005).

## Similarity Hypothesis

The similarity hypothesis states that perceivers empathize with targets similar to themselves, and, as a consequence, subsequent cognitive processing is facilitated. Although all types of similarity—including body posture (Dijkstra et al., 2007), political opinions (Mitchell et al., 2006), and cultural backgrounds (Chiao et al., 2008)—are considered, this paper focuses on similarities in a perceiver’s personality traits (extraversion and neuroticism) and ASD-related characteristics.

The bi-directional white arrow in Figure 1 indicates the interaction between self, or the reader/listener, and the story protagonist during discourse comprehension. The bottom segment of Figure 1 shows the degree of similarity between the readers/listeners and the story protagonist. Readers tend to overestimate the protagonist’s happiness, presumably due to their empathy for characteristics similar to themselves (Komeda et al., 2009). Self (reader) and other (protagonist) overlap when readers are similar to the protagonist and feel empathy for them in a virtual situation (Komeda et al., 2013b). Alternatively, self and other do not overlap when readers (listeners) do not see themselves as similar to the protagonists (speakers) (Komeda et al., 2009, 2013b). In the latter situation, it is difficult to feel empathy (Komeda and Kusumi, 2007). In other words, the degree of overlap represents a possibility for mental simulation (Oatley, 2002; Mar and Oatley, 2008).

The perception of similarity is implicitly evoked via empathy with a target (Stotland, 1969). If the perceiver empathizes with the target, the cognitive processing related to the target is enhanced. For example, reading comprehension is facilitated (e.g., reading accelerates and the outcome-judgment task is performed rapidly and accurately) and memory is enhanced (recognition time is fast and accuracy is better). If the perceiver does not empathize with the target, a perception of dissimilarity is evoked. As a result, cognitive processing is not enhanced (Tversky and Kahneman, 1974; Epley and Gilovich, 2001; Epley et al., 2004).

The next section will discuss empirical evidence obtained from behavioral and neuroimaging studies that supports the similarity hypothesis.

## Evidence Supporting the Similarity Hypothesis

All levels of similarity, from the lowest (e.g., sensation or perception) to the highest (e.g., cognitions regarding politics or social perceptions), are covered by the similarity hypothesis. However, this paper focuses on behavioral and neuroimaging approaches to higher-level cognitive functions.

### Evidence from Behavioral Findings

Similarities in personalities between a reader and characters facilitate reading comprehension (Komeda et al., 2009). Highly extraverted participants judge the outcomes of stories with extraverted protagonists more rapidly than do less extraverted participants, whereas highly neurotic participants judge the outcomes of stories with neurotic protagonists more rapidly than do participants with low levels of neuroticism (Komeda et al., 2013b). Furthermore, a participant’s personality traits predict their empathy for the protagonist: The higher a participant’s extraversion or neuroticism score, the greater their empathy with the extraverted or neurotic protagonist (Komeda et al., 2013b).

### Evidence from Functional Brain Imaging Findings

Discriminating between similar and dissimilar others is performed in the medial prefrontal cortex (mPFC). Activation of the ventral part of the mPFC (vmPFC) is related to processing similar others on a socio–emotional preference task (participants viewed faces with various emotional expressions and made appraisals of whether they liked the face or not) (Chen et al., 2010), judgment of another person’s opinion (Mitchell et al., 2006), and preference for another person’s and their own preference (Tamir and Mitchell, 2010). vmPFC activation during a decision-making task reflects a choice that is executed (no simulation), whereas dmPFC reflects a choice that is modeled but not executed (involving simulation) (Nicolle et al., 2012).

Perceptions of similarity are also based on implicit and automatic processes. According to Lieberman (2007) model of social cognition, the control system (C-system) engages in reflective social cognition (controlled cognitive processing), and the reflexive system (X-system) engages in reflexive social cognition (automatic cognitive processing). The C-system includes the dorsal part of the mPFC (dmPFC), whereas the X- system includes the vmPFC.

Based on the similarity hypothesis, if perceivers empathize with a similar target, the perception of similarity is automatically elicited. These automatic cognitive processes lead to activation of the vmPFC (Mitchell et al., 2006; Jenkins et al., 2008; Komeda et al., 2015). For example, vmPFC activation was significantly greater when making appraisals of self than other (familiar but dissimilar character to the participants, Harry Potter) in TD children and adolescents (Pfeifer et al., 2013).

Alternatively, if perceivers do not empathize with a dissimilar target, a perception of dissimilarity is explicitly elicited. Differences between perceivers and targets are processed explicitly, and the gap between perceivers and targets is resolved through social cognition processes. These effortful cognitive processes lead to activation of the dmPFC (Ferstl and von Cramon, 2002; Ferstl et al., 2005; Mason et al., 2008; Mano et al., 2011). For example, psychophysiological interactions (PPI) analyses showed that dmPFC is a hub of resolutions of social conflict, which is a type of effortful cognitive processes (Watanabe et al., 2014).

## Application of the Similarity Hypothesis to Understand and Support Individuals with ASD

The similarity hypothesis provides the following three predictions to understand the characteristics of individuals with ASD. First, individuals with ASD empathize with others with ASD. Second, individuals with ASD retrieve others with ASD more easily from their memory representation. Third, individuals with ASD support others with ASD.

### Empathy in Individuals with ASD

Although deficits including lack of social reference and difficulty empathizing have been demonstrated in previous studies (Baron-Cohen, 1995; Lombardo et al., 2007; Pfeifer et al., 2013), most target stimuli are oriented at TD individuals. For example, Pfeifer et al. (2013) compared brain activations while making appraisals of one’s self and a familiar but distant other (Harry Potter). Harry Potter do not show a defining characteristics with ASD. As a comparison, brain activations are worth investigating while making appraisal of another character who has the characteristics with ASD. It is probably difficult for individuals with ASD to understand TD individuals, just as it tends to be difficult for TD individuals to understand those with ASD. Komeda et al. (2015) used functional magnetic resonance imaging to examine whether individuals with ASD experience empathy toward other people with ASD. Fifteen high-functioning Japanese participants with ASD (17–41 years of age) and 15 TD Japanese participants (22–40 years of age) matched for age and full and verbal intelligence quotient scores with the participants with ASD were enrolled in this study.

The participants performed social judgment tasks that required reading a sentence related to an autistic character (e.g., “I would rather be alone than with others”) and answering the following question: “Do you agree with the sentence?” Judgments involving a non-autistic character involved reading a sentence (e.g., “Yuya (Japanese male name) would rather be with others than alone”) and answering the following question: “Do you think you are similar to him?”

Interestingly, the results showed that the vmPFC was significantly activated in individuals with ASD in response to characters with ASD and in TD individuals in response to characters without ASD. We found no differences between self- and other-judgments; that is, the vmPFC of individuals with ASD was activated during the self- and other-judgments in response to ASD sentences, whereas the vmPFC of TD individuals was activated during the self- and other-judgments in response to TD sentences. Because the reaction times of other-judgments were longer than those of self-judgments in both the ASD and TD groups, the other-judgments were processed differently from the self-judgments. Nonetheless, it is important that the brain-imaging data showed that the vmPFC was activated during both the self- and other-judgments made by the ASD group in response to ASD sentences and during both the self- and other-judgments made by the TD group in response to TD sentences.

Additionally, higher autism-spectrum quotient scores (Baron-Cohen et al., 2001) in individuals with ASD and TD were significantly correlated with greater activation in the vmPFC while judging characters with ASD traits. Thus, individuals with high levels of ASD traits tend to empathize with others with high levels of ASD, at least on an explicit social judgment task (Komeda et al., 2015).

However, the behavioral results did not support the similarity hypothesis in that the self-reports in the social judgment tasks were not consistent with the diagnostic status of individuals with ASD. Thus, the similarity hypothesis was supported by the implicit measure (vmPFC activation as a physiological measure) but not by the explicit measure (number of agreements as a subjective measure), perhaps in agreement with the distinction between cognitive and emotional empathy (Blair, 2005; Shamay-Tsoory et al., 2005; Saxe, 2006; Völlm et al., 2006; Shamay-Tsoory and Aharon-Peretz, 2007; Jones et al., 2010; Schwenck et al., 2012).

### Memory Retrieval in Individuals with ASD

**Figure 2:**
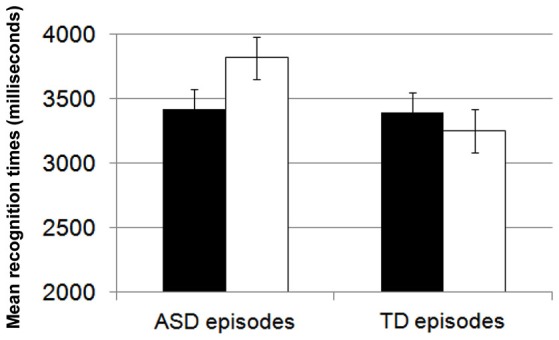
**Comparison of consistent and inconsistent episodes in individuals with ASD**. Black bar shows consistent episodes. White bar shows inconsistent episodes. Individuals with ASD retrieved ASD-consistent episodes faster than they retrieved ASD-inconsistent episodes.

The similarity hypothesis predicts that individuals with ASD should retrieve others with ASD more easily from their memory representation. Based on this hypothesis, I predicted that ASD individuals would demonstrate superior memory for the ASD characters in stories and that TD individuals would demonstrate superior memory for the TD characters in stories. Komeda et al. (2013a) examined differences in episodic memory retrieval between individuals with ASD and TD. Eighteen individuals with ASD (age, 17–40 years) and 17 age- and IQ-matched TD participants (age, 22–40 years) read 24 stories; 12 stories featured protagonists with ASD characteristics, and the other 12 featured TD protagonists. After reading all 24 stories, the participants were asked to complete a recognition task about a target sentence in each story. Although no differences were observed between the ASD and TD groups for encoding processes, they did reveal group differences in memory retrieval. Although individuals with ASD demonstrated the same level of accuracy as did TD individuals, their memory-retrieval patterns differed with respect to response times; individuals with ASD more effectively retrieved ASD-consistent than ASD-inconsistent sentences (Figure 2), and TD individuals retrieved stories with TD more effectively than they retrieved stories with ASD protagonists. Thus, similarities between the reader and the story characters had different effects on memory retrieval in the ASD and TD groups.

### Possibility of ASD Peer Support Based on the Similarity Hypothesis

The similarity hypothesis suggests that individuals with ASD characteristics can help people with ASD. Individuals with ASD can support others with ASD based on empathy toward similar others.

Zercher et al. (2001) examined the effect of peer support using a measure of eye contact and found that participation in an integrated play group was associated with an increase in the joint attention, symbolic play acts, and verbal utterances of two children with ASD. Peer support was provided by a group consisting of 6-year-old boys with ASD and 5-, 6-, and 11-year-old TD boys. As far as I know, scant empirical evidence that children with ASD support other children with ASD is available. Data related to tests of the similarity hypothesis among individuals with ASD may contribute to the effectiveness of self-advocacy groups for these individuals as well as to peer support for children with ASD. For example, Bauminger et al. (2003) reported that many children with ASD reported feelings of loneliness. In this context, children with ASD who have experienced loneliness may be able to support other children with ASD who share similar experiences or feelings.

Finally, the similarity hypothesis is closely related to the “assortative mating” theory, which holds that “like marries like” (Baron-Cohen, 2008). Parents of children who have ASD may not have autism but may have characteristics associated with the condition. Thus, children with ASD have superior attention to detail in terms of perception and memory, and they are strongly attracted to systems (Baron-Cohen, 2006). The assortative mating theory is very useful for thinking about the origins of ASD. However, why “like marries like” occurs remains unclear. The similarity hypothesis can fill the gap. According to the similarity hypothesis, people with ASD empathize with others with ASD. Thus, a preference toward similar others arises. This preference may elicit romantic love and lead to marriage. It will be necessary to investigate genetic backgrounds based on the similarity hypothesis. If the project is completed, we might be able to organize peer (and/or family) support for individuals with ASD based on integrating genetic approaches and similarity-based empathy.

## Conclusions

Behavioral and neuroimaging studies were reviewed based on the similarity hypothesis, which asserts that perceivers empathize with targets similar to themselves. When perceivers empathize with similar targets, a perception of similarity is automatically elicited. This process facilitates cognitive processing, including reading comprehension and memory retrieval. Alternatively, if perceivers do not empathize with dissimilar targets, a perception of dissimilarity is explicitly elicited, and differences between perceivers and targets should be addressed and resolved, as these effortful cognitive processes inhibit cognitive processing.

Potentially, the similarity hypothesis can be applied to the development of educational curricula, such as those for special-needs classes, minority education, and cross-cultural education in order to overcome the effort involved in understanding dissimilar others.

## Conflict of Interest Statement

The author declares that the research was conducted in the absence of any commercial or financial relationships that could be construed as a potential conflict of interest.
